# Mechanical peripheral stimulation for the treatment of gait disorders in patients with Parkinson’s disease: a multi-centre, double-blind, crossover randomized controlled trial

**DOI:** 10.1186/s12984-025-01574-3

**Published:** 2025-04-08

**Authors:** Maria Francesca De Pandis, Carlo Tomino, Stefania Proietti, Rossella Rotondo, Maria Gaglione, Miriam Casali, Massimo Corbo, Lazzaro di Biase, Manuela Galli, Michela Goffredo, Fabrizio Stocchi

**Affiliations:** 1 San Raffaele Hospital Cassino, Via G. Di Biasio, 1, 03043 Cassino, Italy; 2 Department of Human Sciences and Promotion of Quality of Life, San Raffaele Open University, Rome, Italy; 3 IRCCS San Raffaele Roma, Via Della Pisana, 235-00163 Rome, Italy; 4 Casa di Cura del Policlinico Dezza, Milan, Italy; 5 Fondazione Policlinico Universitario Campus Bio-Medico, Operative Research Unit of Neurology, Rome, Italy; 6 Politecnico University, Milan, Italy

**Keywords:** Rehabilitation, AMPS, Gondola^®^ device, Parkinson’s disease, Gait disorders

## Abstract

**Background:**

Pharmacological, surgical and physical therapies ameliorate motor and non-motor symptoms in Parkinson’s disease (PD). Unfortunately, the progression of the disease induces deterioration in daily activities, especially in gait and balance. Invasive and non invasive medical devices have been developed to alleviate drug-resistant symptoms in patients with advanced PD, and automated mechanical peripheral stimulation (AMPS) has been proposed as a new rehabilitative approach.

**Methods:**

This multicentre, double-blind, crossover randomized controlled trial included 83 participants with PD assigned to two groups: AMPS treatment (Gondola^®^ group, n = 40) and placebo treatment (SHAM group, n = 43). The intervention consisted of 6 sessions of stimulation over 3 weeks (AMPS or SHAM), interspersed with a wash-out period of 6 weeks, before switching groups. The aim of this study was to evaluate the effects of AMPS treatment on gait speed and gait-related disorders in subjects with PD.

**Results:**

The Gondola^®^ device resulted in a moderate clinical impact on gait speed in people with PD since the improvement in walking speed exceeded the cut-off of 0.14 m/s in both treatments. The improvement in walking velocity was accompanied by a significantly longer stride length and a prominent increase in % stride length without altering gait cadence in the Gondola^®^ group compared with the SHAM group.

**Conclusions:**

AMPS stimulation improved gait speed in people with PD.

*Trial registration* ClinicalTrials.gov identifier: NCT03843268. Date of registration: 12 Feb 2019, retrospectively registered.

**Supplementary Information:**

The online version contains supplementary material available at 10.1186/s12984-025-01574-3.

## Background

A typical gait pattern characterized by a short-stepped shuffling gait with reduced stride length and walking velocity is commonly recognized in people with Parkinson’s disease (PD) 1, 2. Degeneration of dopaminergic neurons of the basal ganglia and loss of motor control mechanisms lead to movement disorders such as freezing-of-gait (FOG), difficulty turning while walking and postural instability [3–5, affecting patients’ quality of life 3.

Postural disturbances and FOG have limited responsiveness to pharmacological and surgical treatments, probably owing to dysfunctions in sensory‒motor integrations and the involvement of different neurotransmitter pathways and systems 3, 6.

The sole of the foot is a region of the body that is rich in mechanoreceptors, such as the Ruffini corpuscles and the Merkel discs, which are activated by stationary and vibrational pressure stimuli, respectively [7–9. Continuous feedback from this area is particularly relevant during all phases of healthy walking, as it carries critical information regarding the ensemble of movements and adjustments that the brain needs to coordinate with the remaining body to maintain the right posture and produce a fluid walking path 7, 10–13. Similarly, the brain relies on sensory input from the sole of the foot to produce feed forward adaptations, which are important for the control of body posture in response to predictable perturbations 14.

On the basis of this clinical evidence, several studies have investigated the effects of automated mechanical peripheral stimulation (AMPS) on gait and gait-related parameters in a broad range of neurological conditions, including PD 7, 9, 11, 12, 15-17].

This approach, which is commonly implemented with the Gondola^®^ device and textured insoles, has been proven to significantly ameliorate several aspects of gait and balance in patients with PD [18–26].

The conclusive effects of AMPS treatment in clinical practice have not been fully elucidated due to several limitations: (i) the small sample size in most of the studies [19–26]; (ii) the trial design [18, 19]; (iii) the timing of intervention [18–23]; and (iv*)* between-study differences in gait disorder assessments [19, 20, 22, 24, 27] in different PD populations.

Therefore, the aim of this study was to evaluate the effects of AMPS treatment via a Gondola^®^ device on walking speed and gait-related parameters in people with PD in a multicentre, double-blind, crossover randomized controlled study.

## Methods

### Study design

This was a randomized, multicentre, placebo-controlled, double-blind crossover study.

### Allocation and blinding

The participants were randomized into two groups by a computerized system with software designed specifically for this research: one group received effective-AMPS treatment, and the other group received SHAM-AMPS.

The research staff who performed the baseline and follow-up assessments (a neurologist and a neuropsychologist) for each investigational centre, as well as the subjects in the SHAM/effective-AMPS treatment, were unaware of the group assignment and were blinded for the entire study.

### Participants

Participants were recruited between December 2017 and September 2019 from four PD centres located at the IRCCS San Raffaele Roma, San Raffaele Cassino, Casa di Cura of Policlinico Dezza in Milan and Policlinico Universitario Campus Bio-Medico in Rome.

The inclusion criteria were clinically chronic and stable PD (according to the United Kingdom Brain Bank criteria 28), being aged 45 years and older, having an H&Y stage equal to or higher than 2 in the ON state, being able to walk autonomously or with minimal assistance for a 10-m distance in the OFF state, receiving antiparkinsonian treatment at a stable and optimized daily dosage during the 4 weeks prior to the study, and being able to provide informed consent. The exclusion criteria were the presence of any advanced, severe or unstable disease other than PD, which may interfere with the primary and secondary study outcome evaluations (autonomic dysfunction, diabetes, renal or hepatic failure, neoplasia, balance and gait problems of other origin); having a cognitive impairment (Montreal Cognitive Assessment, MoCA < 18); having peripheral neurological or musculoskeletal conditions that may alter balance and/or gait; having no severe lower-limb injuries in the previous 6 months; having no history of neurosurgery (including deep brain stimulation), orthopaedic surgery or epilepsy; having no drug treatment not intended to treat PD that may alter cognitive and/or motor performance; having no history of depression or other psychiatric disorders; and not having severe obesity, defined as a body mass index (BMI) greater than 35.

### Intervention

**Fig. 1:**
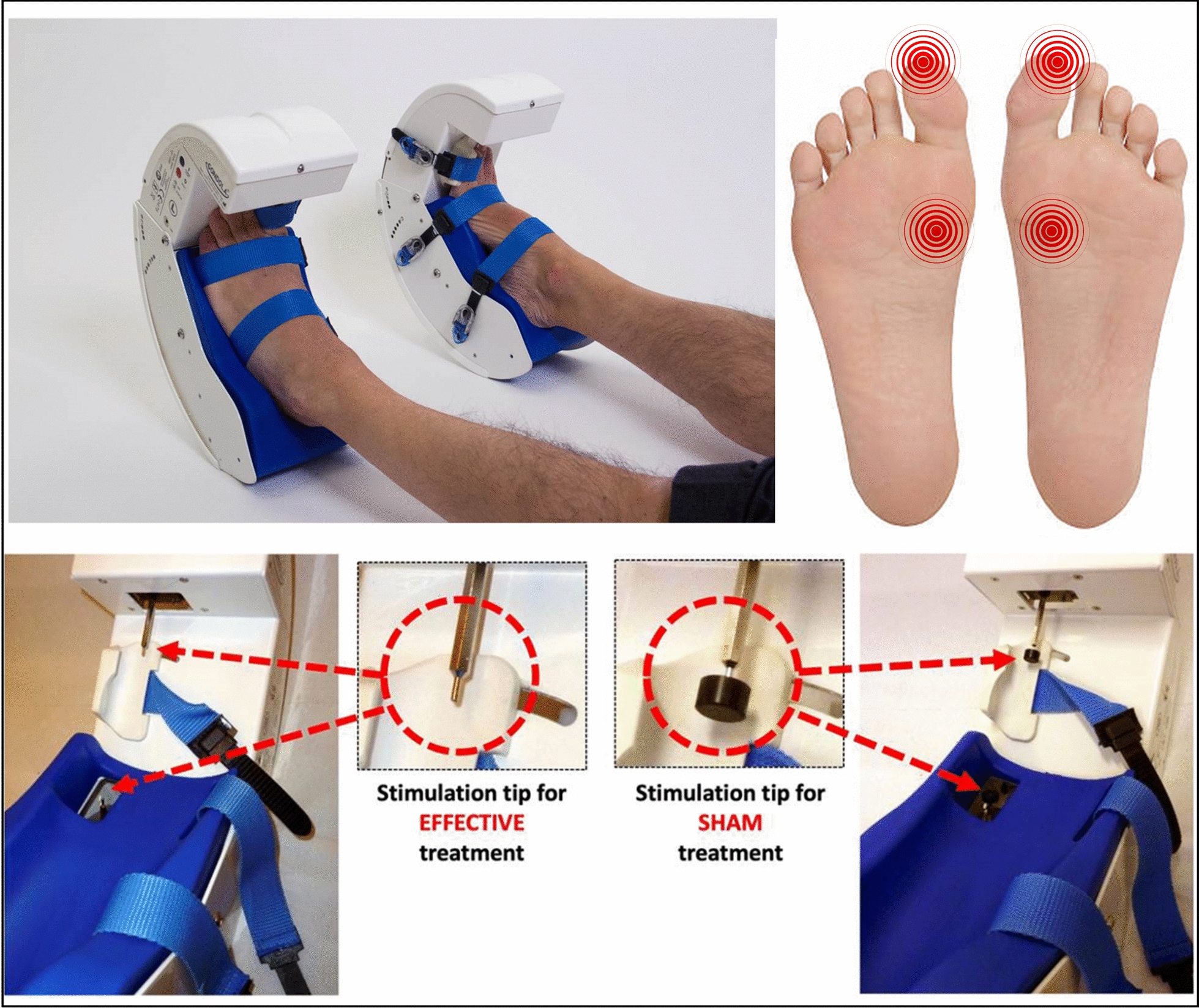
Gondola^®^ medical device. On the left, the effective-AMPS treatment consisted of the application of pressure via rounded stimulation tips in four specific target areas in the patient’s feet; on the right, the placebo stimulation (SHAM-AMPS treatment) was provided using the same device, protocol and therapy cycle used for effective-AMPS, but with a rigid plastic disk with a diameter of 12 mm attached to the stimulation tip

**Fig. 2:**
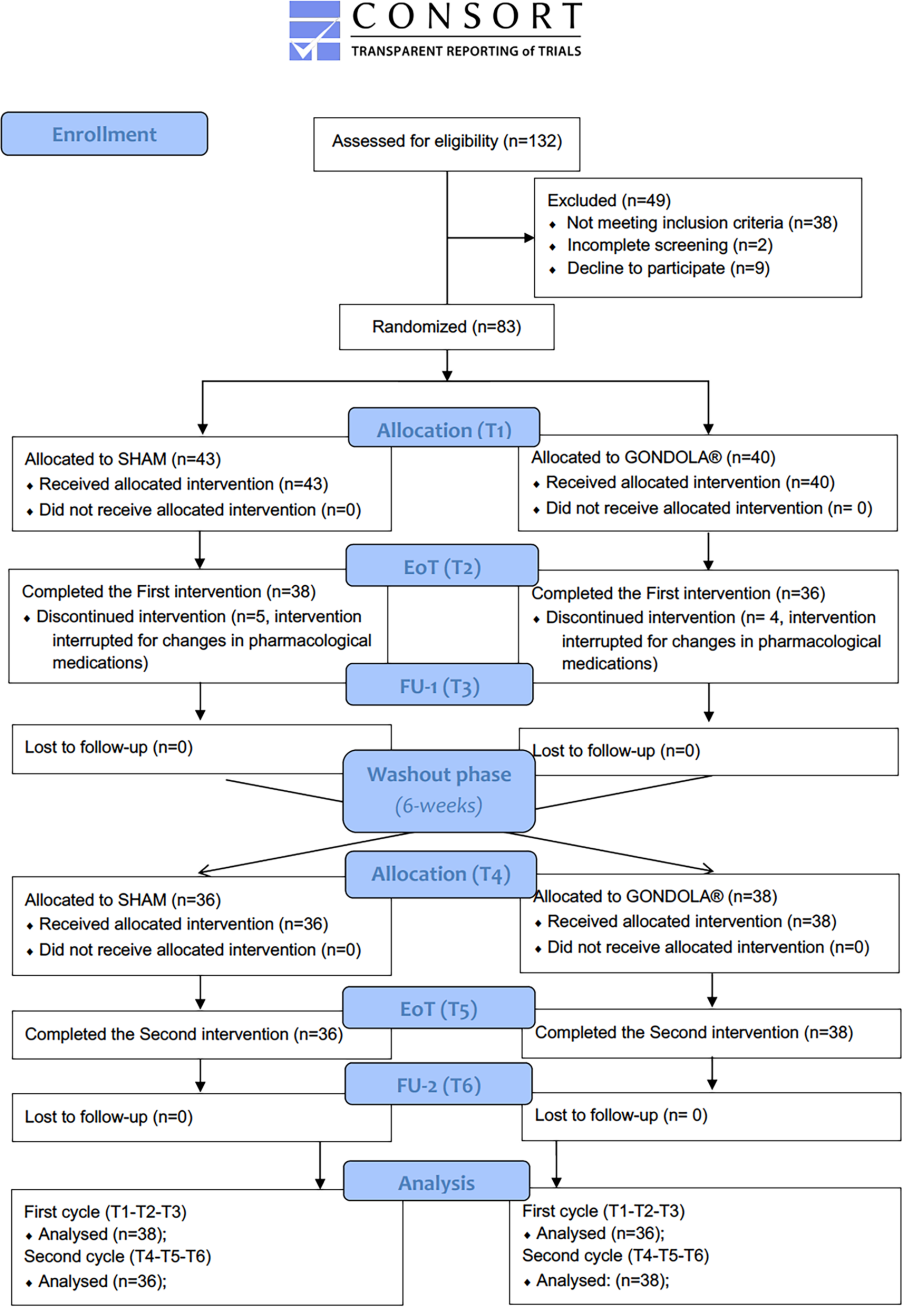
CONSORT flow diagram of the GondoPark study

In accordance with our previous data 19, 21, the “interventions” consisted of 6 sessions of stimulation (AMPS or SHAM) over 3 weeks, interspersed with a wash-out period of 6 weeks. Each “session” consists of 4 cycles, where the “cycle” is intended as the sequential stimulation of the 4 above-indicated target points (2 on each foot, corresponding to the head of the big toe, and the base of the first metatarsal bone between the sesamoid bones, Fig. 1). Specifically, the stimuli were delivered sequentially in the 4 indicated target areas, one after the other—with no intervals between—for 6 s at each target point. Therefore, a single cycle has a duration of 24 s. The overall “treatment” (four sequential cycles) lasts 96 s. A 14-day follow-up evaluation was performed at the end of the intervention (6 sessions of AMPS or SHAM stimulation). Clinical evaluations of the enrolled participants were performed at baseline before the beginning of the intervention (pretreatment, T1), at the end of the first 6 sessions of stimulation (SHAM or AMPS) (T2) and 14 days after the end of the first treatment of the 6 stimulations (T3). The second equivalent intervention of 6 stimulations (SHAM or AMPS) was performed after a 6-week wash-out period (T4, T5 and T6). The CONSORT diagram is shown in Fig. 2.

#### Effective-AMPS

The effective-AMPS treatment consists of the application of pressure via rounded stimulation tips in four specific target areas in the patient’s feet (Fig. 1). To perform this mechanical stimulation, a dedicated medical device (Gondola^®^, Gondola Medical Technologies, Lugano, Switzerland) was used: the system consists of foot supports (left and right) with electrical motors that activate two actuated steel bars with a 2 mm diameter; the motor-activated stimulators apply mechanical pressure in two specific areas of each foot: on the head of the hallux, left and right, and on the 1st metatarsal joint, left and right.

The pressure of stimulation, always applied in the range of 0.3–0.9 N/mm^2^, is set for each subject upon appearance of the monosynaptic reflex in the tibialis anterior muscle by the detection of a preliminary contraction while applying pressure in the contact areas. Once the pressure value has been set using this procedure, the value is recorded to administer the effective-AMPS.

#### SHAM-AMPS

Placebo stimulation was provided using the same device used for effective-AMPS, following the same stimulation protocol and therapy cycle. A rigid plastic disk with a diameter of 12 mm was attached to the stimulation tip; hence, the pressure applied during the treatment decreased as the surface contact increased (Fig. 1). All other steps of the treatment procedure were the same as those for the effective-AMPS treatment.

During the AMPS/SHAMP treatment, the participants were in a lying position.

All tests to assess primary and secondary outcomes were performed at baseline (before the initiation of the GONDOLA^®^/SHAM treatments—T1 and T4), at the end of the 6 stimulations with the GONDOLA^®^/SHAM treatments (EoT, T2 and T5), and 14 days after the end of the GONDOLA^®^/SHAM treatments (follow-up, T3 and T6).

To guarantee blinding, patients were informed that they would take part in the study, the aim of which was to establish the most effective of the two interventional treatments. They were informed that they would receive the same treatment with the same device but with a different distribution of pressure stimulation.

### Adverse events

Before screening, all the staff were trained for safe use of the GONDOLA^®^ medical device by dedicated Gondola Medical Technologies personnel and for the management and reporting of any adverse events.

### Outcomes

The primary outcome of the study was the evaluation of the difference in the change in gait speed from pretreatment to posttreatment between effective-AMPS and SHAM-AMPS with a wearable inertial sensor for motion kinetic parameter assessment (BTS G-WALK system, BTS Bioengineering S.p.A., Italy), with dedicated hardware (BTS G-SENSOR) and software (BTS G-STUDIO) during “OFF” medication periods 29, 30. The wearable inertial sensor was placed at the L5 level by means of a provided elastic belt, with the power connector pointing upwards and the logo facing out. The reference system was correctly defined by keeping the sensor’s position vertical. All the spatial‒temporal gait parameters are provided in the final report. The participants were asked to walk barefoot at a self-selected speed along a flat 10-m walkway. No fewer than four trials were conducted for each session to ensure the repeatability of the measurements.

At least 8 steps for each trial were acquired, among which 6 subsequent steps were considered for each trial; the steps selected were those in the centre of the walkway to assess the participants while they were in the steady-state walking condition, avoiding the initiation and cessation of walking. The gait speed (or walking velocity) [m/s] has been defined as the average instantaneous speed within the gait cycle as an integration of acceleration.

Considering the criteria of Hass et al. which areused to assess clinically relevant changes in gait speed specific to subjects with PD 31, because a significant improvement in gait spe

ed of 0.06 m/s is considered to have a small clinical impact, 0.14 m/s is considered to have a moderate clinical impact, and 0.22 m/s is considered to have a large clinical impact, study success is defined as an improvement in gait speed of no less than 0.06 m/s using the following parameters. We compared the speed values before (V_Pre_) and after (V_Post6_) 6 sessions in the effective-AMPS group (V_PreT_, V_Post6T_) and in the SHAM group (V_PreS_, V_Post6S_).

We considered the speed change between the pre- and post-6 sessions as follows:

$$\Delta {\text{T}}_{{{\text{Treatment}}}} = {\text{V}}_{{{\text{Post}}6{\text{T}}}} {-}{\text{V}}_{{{\text{PreT}}}}$$

$$\Delta {\text{T}}_{{{\text{SHAM}}}} = {\text{V}}_{{{\text{Post}}6{\text{S}}}} - {\text{V}}_{{{\text{preS}}}}$$

where:

V_Post6_ = gait speed after 6 treatment stimulations (at the end of the AMPS/SHAM).

V_pre_ = gait speed before the first treatment stimulation (at the beginning of the AMPS/SHAM).

The efficacy of the treatment was then verified according to the following conditions:

H_0_: ΔT treatment phase–ΔT SHAM phase < 0.06 m/s

vs.

H_a_: ΔT treatment phase–ΔT SHAM phase ≥ 0.06 m/s

The secondary outcome measure of the GondoPark study is the change in the results of the following clinical assessment tools:

(i) Movement Disorder Society-Unified Parkinson's Disease Rating Scale (MDS-UPDRS): a rating tool to follow the longitudinal course of PD;
(ii) Changes in the clinical evaluation of gait-related spatiotemporal parameters by using a wearable device (G-sensor, BTS Bioengineering, Milan):
  - Stride length [m], the distance between two consecutive heel strikes of the same foot;
  - Stride length/height [%], the stride length normalized by the subject height;
  - Cadence [steps/min], the number of steps in a minute;
  - Propulsion [m/s^2^], the anterior‒posterior acceleration peak during the lower-limb swing phase;
(iii) variance in execution time for assessing mobility, balance, walking ability and fall risk (TUG and TUG with dual task, counting while walking);
(iv) change in the clinical evaluation of FOG and dynamic balance assessed using a specific questionnaire (FOG-Q) and a dedicated Mini-Balance Evaluation Systems Test (Mini-BESTest, including 14 items addressing 4 areas of postural control: anticipatory postural adjustments, reactive postural control, sensory orientation, and dynamic gait), respectively.

Participants with PD were in the OFF phase (i.e., having withdrawn from dopaminergic medication overnight) during all the performances to exclude concomitant effects of pharmacological treatments and to attribute the changes, if any, to AMPS treatment only.

### Sample size estimation and statistical analysis

The sample size calculation was estimated on the basis of our previous study 23 by using G*Power software version 3.1.9.1. A sample size of 124 subjects is needed to adequately power this study.

The parameters used to calculate the sample size are as follows:

σ^2^ [variance of the treatment effect in a crossover design] = (W_AA_ + W_BB_ − 2W_AB_ + σ_AA+_σ_BB_), where.

1. W_AA_ = between-patient variance for the pretreatment group (mean velocity ± SD: 0.89 ± 0.27 m/s);
2. W_BB_ = between-patient variance for the posttreatment group (mean velocity ± SD: 1.02 ± 0.23 m/s);
3. W_AB_ = between-patient covariance between the pre- and posttreatment groups;
4. σ_AA_ = within-patient variance for the pretreatment group;
5. σ_BB_ = within-patient variance for the posttreatment group.

Therefore, W_AA_ = 0.27 and W_BB_ = 0.23.

Additional assumptions:

W_AB_ = 0.2 and σ_AA_ = σ_BB_ = 0.1, which yields σ = 0.25.

The sample size estimation is based on a power of 80%, a Type I error rate of 5%, an MCID of 0.06 m/s, an observed treatment effect of 0.14 m/s, and a treatment effect variance of σ = 0.25.

An attrition rate of 6% was considered in addition to the sample size calculated; therefore, a total sample size of 132 participants with PD was considered necessary for this study.

A post hoc power analysis was conducted to retest the adequacy of the achieved sample size by using G*Power software version 3.1.9.7, estimating the effect size on the basis of the first outcome results and a Type I error rate of 5%.

For statistical analysis, after testing for normality via the Kolmogorov test, the normally distributed quantitative variables were expressed as the means and standard deviations; the qualitative variables were expressed as percentages. The comparisons between and within groups were made with an unpaired t test with Bonferroni correction for multiple comparisons, and the α level was adjusted for all the comparisons (n = 15). The associations between categorical variables were tested using the chi-square test. The data were analysed using a time × treatment multivariate general linear model (GLM). The p values from multivariate tests are reported to describe the time (first/second intervention) × treatment (sequence AMPS/SHAM or SHAM/AMPS) interaction effects of the variables analysed. Patients were considered random effects since a randomization process for AMPS/SHAM treatment was applied. The partial eta-squared (η2) effect size is reported considering that the magnitude of the effect was defined as 0.01 = small, 0.06 = medium, 0.13 = large 31, 32.

SPSS Statistics 28.0 and R 4.2.2 were used, and the data were considered statistically significant when p < 0.003.

### Ethics

The trial was conducted in accordance with the Declaration of Helsinki (October 1996) and the International Conference on Harmonization Guidelines on Good Clinical Practice (GCP) (CPMP/ICH/135/95).

The study has been registered at: clinicaltrials.gov (https://clinicaltrials.gov/), NCT03843268, with the alternative name of GondoPark V2. The study was approved by the Ethics Committee of the Coordination Center IRCCS San Raffaele Rome on 19 April 2017 (verbale 4/2017- Registro Pareri E/10/17) and by each local institutional IRB/IEC. Signed informed consent was obtained from each patient before any study procedure.

## Results

### Participants

Among 132 subjects with PD screened for eligibility in the GondoPark study, 38 did not meet the inclusion criteria, 2 did not complete the screening, and 9 declined to participate (n = 2); therefore, 49 individuals with PD were excluded, and 83 participants were randomized into 2 groups.

One group started with AMPS treatment (Gondola^®^ group, n = 40), and the other started with placebo treatment (n = 43, SHAM group). At the end of the first intervention (EoT, T2), 5 participants in the SHAM group and 4 patients in the Gondola^®^ group interrupted the intervention for changes in pharmacological treatments and were discontinued. Therefore, 38 participants in the SHAM group and 36 participants in the Gondola^®^ group completed the first intervention of 6 sessions of stimulation before switching among groups, after a wash-out period of 6 weeks.

All participants completed the second intervention (n = 36 in the SHAM group and n = 38 in the Gondola^®^ group; EoT, T5), as shown in the CONSORT flow diagram (Fig. 2).

### Baseline characteristics

The participants’ demographic characteristics and baseline scores for the primary and secondary outcomes are reported in Table 1. The participants were well distributed between the two groups in terms of sex, age and BMI. Global cognitive functions were relatively preserved, as indicated by the MoCA results (score > 17.54); all the subscales of the MDS-UPDRS (parts I, II, III and IV) revealed moderate disease severity. The majority of the subjects with PD had a moderate to severe disease stage (H&Y ≥ 3), postural instability and FOG, as indicated by the Mini-BESTest and FOG-Q, respectively (Table 1).

**Table 1:** Clinical characteristics of participants with PD at baseline (T1)

Variables	SHAM (n = 38)	Gondola^®^ (n = 36)	p value
Sex, n male/female (%)	20/18 (52.6/47.4)	19/17 (52.8/47.2)	1.000
Age (years)	70.2 ± 10.3	69.2 ± 7.3	0.437
BMI (kg/m^2^)	27.8 ± 4.7	27.7 ± 4.0	0.896
MoCA (0–30)	24.7 ± 2.8	24.0 ± 2.8	0.301
MDS-UPDRS I (phase OFF,0–52 points)	13.8 ± 5.1	12.7 ± 6.4	0.903
MDS-UPDRS II (phase OFF, 0–52 points)	18.6 ± 7.4	18.4 ± 7.8	0.821
MDS-UPDRS III (phase OFF, 0–132 points)	52.8 ± 15.8	51.9 ± 14.9	0.595
MDS-UPDRS IV (phase OFF, 0–24 points)	5.2 ± 2.6	4.9 ± 2.6	0.504
H&Y stage, n (%)			
− 2	7 (18.4)	6 (16.7)	0.438
− 2.5	6 (15.8)	5 (13.9)	
− 3	16 (42.1)	21 (58.3)	
− 4	9 (23.7)	4 (11.1)	
FOG-Q (0–24)	10.6 ± 4.7	11.3 ± 4.9	0.504
Mini-BESTest (0–28)	14.8 ± 5.2	15.3 ± 4.5	0.580

The values are the means ± SDs, except for sex and H&Y score (number of patients followed by percentage). For statistical analysis, an unpaired t test was used. A p value < 0.05 was considered toindicate statistical significance. *BMI* body mass index, *MoCA* Montreal Cognitive Assessment, *MDS-UPDRS* Movement Disorder Society-Unified Parkinson’s Disease Rating Scale (part I–Nonmotor aspects of experiences of daily living; part II–Motor aspects of experiences of daily living; part III–Motor examination; part IV- Motor complications), *H&Y* Hoehn and Yahr (2 = Bilateral involvement without impairment of balance; 2.5 = Mild bilateral disease with recovery on pull test; 3 = Mild to moderate bilateral disease; some postural instability; physically independent; 4 = Severe disability; still able to walk or stand unassisted); *FOG-Q* Freezing Of Gait–Questionnaire, *Mini-BESTest* Mini-Balance Evaluation Systems Test

### Outcomes

Study success, as described in the methods section, was achieved. Indeed, velocity variation (ΔT = V_Post6_–V_Pre_) in the first (T2-T1) and second interventions (T5-T4) satisfied the alternative hypothesis (respectively, Δ(T2-T1) _Gondola_: 0.19 m/s *vs.* Δ(T2-T1) _SHAM_: 0.04 m/s; Δ(T5-T4) _Gondola®_: 0.14 m/s *vs.* Δ(T2-T1) _SHAM_: 0.02 m/s). Since treatment with the Gondola^®^ device led to an improvement in velocity of no less than 0.06 m/s for both interventions, the null hypothesis can be rejected. Furthermore, considering the criteria of Hass et al. [31], changes in gait speed in people with PD treated with the Gondola^®^ device were clinically relevant since a moderate improvement in walking speed of 0.14 m/s was reached. Although the number of analysed participants did not reach the required sample size, the large effect size still led to a post hoc power of 95.6% (alpha = 0.05).

To investigate the factors influencing the change in walking speed, several spatiotemporal features of gait were investigated in the first and second interventions, as shown in Tables 2 and 3.

**Table 2:** Spatiotemporal parameters of gait analysis and functional motor assessment of participants with PD (Sham vs. Gondola®) at first intervention (6 sessions of stimulation) (T1-T2)

		SHAM (n = 38)	Gondola® (n = 36)	SHAM (n = 38)	Gondola® (n = 36)	SHAM (n = 38)	Gondola® (n = 36)	p value
		T1		T2		T2 *vs*T1		
Primary outcome								
Velocity [m/s]		0.62 ± 0.19	0.68 ± 0.21	0.66 ± 0.21	0.87 ± 0.24	0.04 ± 0.13	0.19 ± 0.21	** < 0.001**
Secondary outcomes								
Spatiotemporal parameters of gait analysis	Cadence [stride/min]	103.15 ± 15.33	103.04 ± 14.60	103.95 ± 15.66	107.48 ± 13.79	0.79 ± 16.97	4.44 ± 9.74	0.271
	Stride length [m]	0.75 ± 0.23	0.80 ± 0.22	0.78 ± 0.22	0.98 ± 0.23	0.03 ± 0.17	0.18 ± 0.22	**0.002**
	% Stride length	46.46 ± 14.03	49.07 ± 12.49	48.32 ± 13.81	59.84 ± 12.06	1.86 ± 10.42	10.77 ± 13.58	**0.003**
	Stride duration [s]	1.21 ± 0.16	1.21 ± 0.19	1.20 ± 0.18	1.15 ± 0.21	− 0.012 ± 0.18	− 0.059 ± 0.12	0.212
	Stance phase [%]	61.94 ± 2.53	61.54 ± 2.76	61.57 ± 2.61	60.87 ± 2.60	− 0.376 ± 2.91	− 0.672 ± 2.78	0.660
	Swing phase [%]	38.05 ± 2.53	38.46 ± 2.76	38.43 ± 2.61	39.13 ± 2.60	0.376 ± 2.91	0.672 ± 2.78	0.660
	Initial phase of double support [%]	11.83 ± 2.59	11.54 ± 2.75	11.40 ± 2.62	10.84 ± 2.48	− 0.424 ± 2.97	− 0.70 ± 2.85	0.687
	Single support phase [%]	38.21 ± 2.66	38.44 ± 2.73	38.54 ± 2.61	39.17 ± 2.53	0.336 ± 2.94	0.732 ± 2.81	0.562
	Propulsion [m/s^2^]	3.76 ± 1.34	3.85 ± 1.33	3.81 ± 1.43	4.64 ± 1.43	0.045 ± 1.11	0.793 ± 1.51	0.019
Functional motor assessments	FOG-Q (0–24)	10.62 ± 4.80	11.76 ± 4.69	10.52 ± 4.73	10.32 ± 4.46	− 0.11 ± 1.52	− 1.44 ± 2.27	0.005
	TUG [s]	22.40 ± 8.36	25.54 ± 17.11	22.94 ± 10.24	22.60 ± 24.92	0.54 ± 6.72	− 2.94 ± 11.59	0.121
	TUG Dual-task [s]	28.97 ± 11.94	39.71 ± 53.49	31.97 ± 16.44	35.86 ± 55.22	3.00 ± 13.15	− 3.85 ± 20.96	0.099
	Mini-BESTest (0–28)	14.83 ± 5.21	15.34 ± 4.46	14.76 ± 4.98	16.14 ± 4.42	− 0.08 ± 1.23	0.80 ± 1.54	0.009
	MDS-UPDRS							
	− 2.12	1.73 ± 0.92	1.83 ± 0.89	1.67 ± 0.94	1.65 ± 0.87	− 0.05 ± 0.57	− 0.17 ± 0.51	0.365
	− 2.13	1.32 ± 1.04	1.60 ± 1.00	1.29 ± 1.02	1.34 ± 1.00	− 0.027 ± 0.50	− 0.26 ± 0.61	0.084
	− 3.10	2.19 ± 0.83	2.03 ± 0.77	2.16 ± 0.87	1.67 ± 0.76	− 0.027 ± 0.37	− 0.36 ± 0.60	0.006
	− 3.11	1.35 ± 1.25	1.36 ± 1.06	1.35 ± 1.18	1.00 ± 0.98	0.01 ± 0.47	− 0.36 ± 0.65	0.009
	− 3.12	2.00 ± 1.00	2.03 ± 1.12	2.13 ± 1.03	1.67 ± 1.21	0.13 ± 0.42	− 0.36 ± 0.49	** < 0.001**

The values are the means ± SDs. For statistical analysis, an unpaired t test with Bonferroni correction was used for multiple comparisons (n = 15). A p value < 0.003 was considered toindicate statistical significance

*MDS-UPDRS* Movement Disorder Society-Unified Parkinson’s Disease Rating Scale (part II–Motor aspects of experiences of daily living: 2.12 WALKING AND BALANCE; 2.13 FREEZING; part III–Motor examination: 3.10 GAIT; 3.11 FREEZING OF GAIT; 3.12 POSTURAL STABILITY), *FOG-Q* Freezing Of Gait–Questionnaire, *TUG* Timed up-and-Go, *Mini-BESTest* Mini-Balance Evaluation Systems Test

**Table 3:** Spatiotemporal parameters of gait analysis and functional motor assessment of participants with PD (Sham *vs.* Gondola®) at second intervention (6 sessions of stimulation) (T4-T5)

		SHAM (n = 36)	Gondola® (n = 38)	SHAM (n = 36)	Gondola® (n = 38)	SHAM (n = 36)	Gondola® (n = 38)	p value
		T4		T5		T5 *vs.* T4		
Primary outcome								
Velocity [m/s]		0.73 ± 0.25	0.61 ± 0.20	0.76 ± 0.24	0.76 ± 0.22	0.02 ± 0.10	0.14 ± 0.17	** < 0.001**
Secondary outcome								
Spatiotemporal parameters of gait analysis	Cadence [stride/min]	105.57 ± 18.85	103.55 ± 14.81	105.67 ± 16.49	103.40 ± 12.70	0.11 ± 9.04	− 0.15 ± 9.74	0.906
	Stride length [m]							
	Stride length	0.85 ± 0.25	0.74 ± 0.23	0.88 ± 0.23	0.88 ± 0.21	0.03 ± 0.11	0.15 ± 0.18	** < 0.001**
	% Stride length	51.69 ± 14.32	45.54 ± 14.27	53.32 ± 12.94	55.97 ± 14.66	1.63 ± 6.54	10.42 ± 12.28	** < 0.001**
	Stride duration [s]	1.20 ± 0.28	1.21 ± 0.18	1.20 ± 0.28	1.20 ± 0.16	− 0.001 ± 0.09	-0.006 ± 0.12	0.843
	Stance phase [%]	61.22 ± 1.98	61.68 ± 2.14	61.11 ± 2.18	60.87 ± 1.91	− 0.12 ± 2.06	-0.81 ± 2.37	0.182
	Swing phase [%]	38.77 ± 1.98	38.31 ± 2.14	38.89 ± 2.18	39.13 ± 1.91	0.12 ± 2.06	0.82 ± 2.38	0.182
	Initial phase of double support [%]	11.25 ± 1.96	11.62 ± 2.15	11.04 ± 2.13	10.79 ± 1.89	− 0.20 ± 2.03	-0.82 ± 2.38	0.233
	Single support phase [%]	38.62 ± 1.93	38.32 ± 2.11	38.95 ± 2.15	39.20 ± 1.91	0.32 ± 2.07	0.87 ± 2.43	0.303
	Propulsion [m/s^2^]	3.99 ± 1.25	3.42 ± 1.27	4.10 ± 1.40	4.03 ± 1.30	0.11 ± 0.67	0.61 ± 1.04	0.018
Functional motor assessments	FOG-Q (0–24)	10.85 ± 4.46	10.96 ± 5.15	10.71 ± 4.26	9.87 ± 4.59	− 0.14 ± 0.75	− 1.09 ± 1.80	0.012
	TUG [s]	22.96 ± 9.84	27.21 ± 23.53	21.32 ± 9.02	20.25 ± 9.07	− 1.64 ± 4.79	− 6.98 ± 16.70	0.109
	TUG Dual-task [s]	27.39 ± 13.92	37.15 ± 36.39	29.64 ± 18.76	31.34 ± 29.57	2.25 ± 16.89	− 5.81 ± 16.40	0.066
	Mini-BESTest (0–28)	15.42 ± 4.38	14.71 ± 5.79	15.92 ± 4.52	15.93 ± 5.45	0.50 ± 2.06	1.21 ± 1.98	0.174
	MDS-UPDRS							
	− 2.12	1.71 ± 0.81	1.72 ± 0.89	1.67 ± 0.81	1.69 ± 0.93	− 0.03 ± 0.19	− 0.03 ± 0.47	0.963
	− 2.13	1.64 ± 1.06	1.47 ± 1.13	1.57 ± 1.03	1.28 ± 0.99	− 0.07 ± 0.26	− 0.18 ± 0.39	0.193
	− 3.10	1.82 ± 0.72	2.15 ± 0.92	1.75 ± 0.70	1.87 ± 0.79	− 0.07 ± 0.26	− 0.28 ± 0.52	0.060
	− 3.11	1.39 ± 1.10	1.62 ± 1.23	1.35 ± 1.06	1.22 ± 1.12	− 0.03 ± 0.57	− 0.40 ± 0.50	0.010
	− 3.12	1.96 ± 1.10	2.09 ± 1.09	1.89 ± 0.99	1.90 ± 0.99	− 0.07 ± 0.71	− 0.18 ± 0.53	0.477

The values are the means ± SDs. For statistical analysis, an unpaired t test with Bonferroni correction was used for multiple comparisons (n = 15). A p value < 0.003 was considered toindicate statistical significance

*MDS-UPDRS* Movement Disorder Society-Unified Parkinson’s Disease Rating Scale (part II–Motor aspects of experiences of daily living: 2.12 WALKING AND BALANCE; 2.13 FREEZING; part III–Motor examination: 3.10 GAIT; 3.11 FREEZING OF GAIT; 3.12 POSTURAL STABILITY), *FOG-Q* Freezing Of Gait–Questionnaire, *TUG* Timed up-and-Go, *Mini-BESTest* Mini-Balance Evaluation Systems Test

A comparison of gait analysis between the two groups revealed that participants with PD after Gondola® treatment presented a significantly longer stride length and a prominent increase in % stride length, which were ~ 5.8– and ~ 6.4 times greater than those in the SHAM group. Increases in walking speed and stride length did not affect gait cadence (Tables 2 and 3).

The effects of Gondola^®^ treatment with respect to SHAM treatment on motor function were investigated. Except for preliminary evidence of improvement in postural instability (MDS-UPDRS item 3.12) in the first intervention (6 sessions of stimulation) of Gondola^®^-treatment compared with the control group, the data did not reveal significant changes in the motor-functional secondary outcomes.

Notably, two-way ANOVA, considering the time (first/second intervention) × treatment (sequence AMPS/SHAM or SHAM/AMPS) interaction effects, revealed that carry-over effects from the first to second intervention cannot be excluded with respect to changes in velocity, stride duration and length, stride length, stance phase and propulsion (Supplementary material).

## Discussion

This multicentric, double-blinded, randomized controlled clinical trial revealed the effects of AMPS treatment on improving walking speed and gait-related parameters, such as absolute stride length and %stride length, in subjects with PD. The effects were comparable for both interventions, as shown in Tables 2 and 3.

The analysis of variance revealed time (first/second intervention) × treatment (sequence AMPS/SHAM or SHAM/AMPS) interaction effects on several variables, including walking speed. The long-term effects of AMPS treatments were investigated in our previous study, which revealed that AMPS treatment appears to improve gait parameters, restore rhythmicity, and reduce the risk of falls 19. Although the benefits were maintained for up to 10 days after the last treatment, a progressive reduction in walking speed was still detected compared with that at baseline. The data reported here surprisingly show that the long-term effects of AMPS treatment may be greater than expected and that a 6-week wash-out period is not sufficient to completely abolish the stimulation benefits.

Therefore, the study has several intrinsic limitations: long-term effects of AMPS stimulation should be assessed with longer follow-up periods to establish a proper wash-out period, thus excluding any possible interaction effects. Since the main endpoint of the GondoPark study is a change in gait speed assessed in the OFF condition, further studies are needed to definitively establish the clinical relevance of AMPS treatment in subjects with PD in the ON phase. The strong effect of drugs can shadow the change obtained in the OFF condition; future clinical trials will compare the effects of dopaminergic treatment alone with those of dopaminergic treatment plus AMPS therapy.

The decrease in OFF severity, as revealed by improvements in gait and gait-related parameters in subjects with PD treated with AMPS with respect to SHAM, represents a prerequisite for testing the effect of AMPS treatment in a sample of severely impaired patients, who do not derive significant benefits from drug schedule adjustment. The study should also include an assessment of the impact of AMPS therapy on patient care in terms of quality of life and the number of falls.

## Conclusions

This multi-centre, double-blind, crossover randomized controlled trial confirmed that automated mechanical peripheral stimulation of the soles of the feet resulted in a moderate clinical impact on gait speed in a large population of people with PD.

## Supplementary Information

Supplementary material 1.

## Data Availability

No datasets were generated or analysed during the current study.

## Abbreviations

PD: Parkinson’s disease
AMPS: Automated mechanical peripheral stimulation
FOG: Freezing of gait
H&Y: Hoehn and Yahr
EoT: End-of-Treatment
FOG-Q: Freezing of Gait Questionnaire
Mini-BESTest: Mini Balance Evaluation Systems Test
ICH: International Conference on Harmonization Guidelines
GCP: Good Clinical Practice
BMI: Body mass index
MoCA: Montreal Cognitive Assessment
MDS-UPDRS: Movement Disorder Society-Unified Parkinson’s Disease Rating Scale
TUG: Timed up-and-Go
