# Supplementary material for: Mechanical peripheral stimulation for the treatment of gait disorders in patients with Parkinson’s disease: a multi-centre, double-blind, crossover randomized controlled trial
Source: J Neuroeng Rehabil. 2025 Apr 8;22:77. doi: 10.1186/s12984-025-01574-3 (PMC11978199; doi:10.1186/s12984-025-01574-3)
Supplement: Supplementary file 1 — Supplementary material 1. [file 12984_2025_1574_MOESM1_ESM.docx]

**ANOVA using GLM**

Data were analyzed using a treatment x time point general multivariate linear model (GLM). Thep-value from multivariate tests are reported to describe overall time (period), treatment (sequence) and treatment (sequence) x time interaction effects of variables analyzed.

The partial eta-squared (η2) effect size are reported considering the magnitude of effect was defined as 0.01 = small, 0.06 = medium, 0.13 = large*

* [Zhang Y, Huo M, Zhou J, Xie S. PKSolver: an add-in program for pharmacokinetic and pharmacodynamic data analysis in Microsoft excel. Comput Methods Prog Biomed. 2010;99(3):306–314. doi: 10.1016/j.cmpb.2010.01.007.

Pruessner JC, Kirschbaum C, Meinlschmid G, Hellhammer DH. Two formulas for computation of the area under the curve represent measures of total hormone concentration versus time-dependent change. Psychoneuroendocrinology. 2003;28(7):916–931. doi: 10.1016/S0306-4530(02)00108-7].

Sequence represents Gondola →Sham or Sham→ Gondola;

Subjectsequal patients,

Period (time) represents the first cycle (1) or the second cycle (2).

*Tables . Analysis of Variance Table for a Standard 2 × 2 Crossover Design*

**Variable:** ΔVelocity [m/s]

Source of variation d.f. Type III sum of squares Mean square F P value η^2^

Model 4 0.774 0.194 7.495 0.0001 0.175

Period 1 0.042 0.042 1.622 0.205 0.011

Sequence 1 0.012 0.012 0.448 0.504 0.003

Period*Sequence 1 0.644 0.644 24.958 **0.0001** 0.150

Subjects 1 0.076 0.076 2.928 0.089 0.020

Error 141 3.641 0.026

Total 146 5.829

R-quadrato = ,175 (R-quadrato adattato = ,152)

**Variable:** ΔStride duration [s]

Source of variation d.f. Type III sum of squares Mean square F P value η^2^

Model 4 392,270 98,067 4.825 0.001 0.120

Period 1 25.022 25.022 1.231 0.269 0.009

Sequence 1 0.497 0.497 0.024 0.876 0.000

Period*Sequence 1 360.737 360.737 17.748 **0.0001** 0.112

Subjects 1 8.739 8.739 0.430 0.513 0.003

Error 141 2865.883 20.325

Total 146 3940.281

R-quadrato = ,120 (R-quadrato adattato = ,095)

**Variable:** Δ Cadence [stride/min]

Source of variation d.f. Type III sum of squares Mean square F P value η^2^

Model 4 617.989 154.497 1.098 0.360 0.030

Period 1 256.185 256.485 1.820 0.179 0.013

Sequence 1 127.785 127.785 0.908 0.342 0.006

Period*Sequence 1 99.519 99.519 0.707 0.402 0.005

Subjects 1 132.428 132.428 0.941 0.334 0.007

Error 141 19844.351 140.740

Total 146 20691.742

R-quadrato = ,030 (R-quadrato adattato = ,003)

**Variable:** Δ Stride length [m]

Source of variation d.f. Type III sum of squares Mean square F P value η^2^

Model 4 0.721 0.180 5.800 0.0001 0.141

Period 1 0.010 0.010 0.309 0.579 0.002

Sequence 1 0.007 0.007 0.215 0.644 0.002

Period*Sequence 1 0.663 0.663 21.351 **0.0001** 0.132

Subjects 1 0.039 0.039 1.242 0.267 0.009

Error 141 4.380 0.031

Total 146 6.418

R-quadrato = ,141 (R-quadrato adattato = ,117)

**Variable:** Δ % Stride length

Source of variation d.f. Type III sum of squares Mean square F P value η^2^

Model 4 3116,455 779.114 6.459 0.0001 0.155

Period 1 3,387 3,387 0.028 0. 867 0.000

Sequence 1 ,102 ,102 ,001 0.977 0.000

Period*Sequence 1 2818,871 2818,871 23,369 **0.0001** 0.142

Subjects 1 259,808 259,808 2,154 0.144 0.015

Error 141 17007,802 120,623

Total 146 25681,700

R-quadrato = ,155 (R-quadrato adattato = ,131)

**Variable:** Δ Stance phase [%]

Source of variation d.f. Type III sum of squares Mean square F P value η^2^

Model 4 12,748 3,187 0.486 0.746 0.014

Period 1 0.122 0.122 0.028 0. 892 0.000

Sequence 1 1.624 1.624 1.357 0. 246 0.010

Period*Sequence 1 8.903 8.903 23.369 **0.0001**  0.142

Subjects 1 2.011 2.011 0.306 0.581 0.002

Error 141 925.155 6,561

Total 146 974.133

R-quadrato = ,014 (R-quadrato adattato =- ,014)

**Variable:** Δ Swing phase [%]

Source of variation d.f. Type III sum of squares Mean square F P value η^2^

Model 4 12.752 3.188 0.486 0.746 0.014

Period 1 0.113 0.113 0.017 0. 896 0.000

Sequence 1 1.631 1.631 0.249 0. 619 0.002

Period*Sequence 1 8.901 8.901 1.357 0.246 0.010

Subjects 1 2.017 2.017 0.307 0.580 0.002

Error 141 925.071 6,561

Total 146 974.253

R-quadrato = ,014 (R-quadrato adattato =- ,014)

**Variable:** ΔInitial phase of double support [%]

Source of variation d.f. Type III sum of squares Mean square F P value η^2^

Model 4 14,246 3,562 0. 531 0.713 0.015

Period 1 0.089 0.089 0.013 0. 908 0.000

Sequence 1 1.291 1.291 0.192 0. 662 0.001

Period*Sequence 1 7.130 7.130 1.062 0.304 0.007

Subjects 1 5.640 5.640 0.840 0.361 0.006

Error 141 946.280 6,711

Total 146 1003.501

R-quadrato = ,015 (R-quadrato adattato =- ,013)

**Variable:** Δ Single support phase [%]

Source of variation d.f. Type III sum of squares Mean square F P value η^2^

Model 4 12,815 3.204 0.477 0.753 0.013

Period 1 0.139 0.139 0.021 0. 886 0.000

Sequence 1 0.295 0.295 0.044 0. 834 0.000

Period*Sequence 1 7.856 7.856 1.170 0.281 0.008

Subjects 1 4.248 4.248 0.633 0.248 0.004

Error 141 946.862 6,715

Total 146 1006.884

R-quadrato = ,015 (R-quadrato adattato =- ,013)

**Variable:** ΔPropulsion [m/ s^2^]

Source of variation d.f. Type III sum of squares Mean square F P value η^2^

Model 4 17.186 4.297 3.430 0.010 0.089

Period 1 0.127 0.127 0.101 0. 751 0.001

Sequence 1 0.472 0.472 0.377 0. 540 0.003

Period*Sequence 1 13.918 13.918 11.112 **0.001**  0.073

Subjects 1 2.463 2.463 1.966 0.163 0.014

Error 141 176.605 1.253

Total 146 215,897

R-quadrato = ,089 (R-quadrato adattato = ,063)

Table 4. - Spatio-temporal parameters of gait analysis and functional motor assessment of participants with PD (Sham vs Gondola®) at follow-up (T3 and T6)

|  | | **SHAM**  **(n= 36)** | **Gondola® (n=38)** | **p-value** | **SHAM**  **(n= 36)** | **Gondola® (n=38)** | **p-value** |
| --- | --- | --- | --- | --- | --- | --- | --- |
|  | | **T3** | |  | **T6** | |  |
| **Primary outcome** | |  |  |  |  |  |  |
| Velocity [m/s] | | 0.64±0.20 | 0.78±0.23 | 0.009 | 0.76±0.28 | 0.65±0.18 | 0.052 |
| **Secondary outcome** | |  |  |  |  |  |  |
| Spatiotemporal parameters of gait analysis | Cadence [stride/min] | 102.08±14.5 | 103.9±15.6 | 0.596 | 103.94±15.6 | 102.08±14.52 | 0.156 |
|  | Stride length [m] | 0.77±0.21 | 0.90±0.18 | 0.007 | 0.87±0.25 | 1.03±1.57 | 0.519 |
|  | % Stride length | 48.04±13.12 | 50.95±9.92 | 0.013 | 52.69±14.02 | 49.46±14.20 | 0.328 |
|  | Stride duration [s] | 1.22±0.18 | 1.21±0.28 | 0.870 | 1.19±0.29 | 1.44±1.31 | 0.285 |
|  | Stance phase [%] | 61.79±1.87 | 61.09±2.28 | 0.153 | 60.69±2.14 | 61.03±2.33 | 0.504 |
|  | Swing phase [%] | 38.21±1.86 | 38.91±2.27 | 0.153 | 39.31±2.14 | 38.78±2.15 | 0.295 |
|  | Initial phase of double support [%] | 11.69±1.91 | 11.03±2.23 | 0.173 | 10.61±2.13 | 11.08±2.15 | 0.346 |
|  | Single support phase [%] | 38.34±1.95 | 39.01±2.23 | 0.175 | 39.38±2.16 | 38.86±2.19 | 0.315 |
|  | Propulsion [m/s^2^] | 3.60±1.31 | 4.19±1.30 | 0.056 | 4.16±1.65 | 3.54±1.19 | 0.069 |
|  |  |  |  |  |  |  |  |
| Functional motor assessments | FOG-Q (0-24) | 11.06±4.64 | 10.22±4.90 | 0.475 | 10.57±4.36 | 9.97±4.82 | 0.617 |
|  | TUG [s] | 25.45±18.85 | 20.57±13.09 | 0.222 | 22.89±19.5 | 21.03±8.28 | 0.635 |
|  | TUG Dual-task [s] | 37.0.3±48.3 | 35.09±55.49 | 0.476 | 26.07±15.51 | 31.83±21.64 | 0.266 |
|  | MINIBEST (0-28) | 14.71±5.33 | 16.06±4.55 | 0.268 | 15.75±4.43 | 15.5±5.36 | 0.848 |
|  | MDS-UPDRS   - 2.12 - 2.13 - 3.10 - 3.11 - 3.12 | 1.74±1.01  1.45±1.09  2.11±0.87  1.37±1.21  2.11±1.08 | 1.67±0.89  1.46±1.09  1.75±0.75  1.21±0.99  1.84±1.06 | 0.743  0.992  0.075  0.557  0.310 | 1.71±0.90  1.57±1.06  1.93±0.81  1.43±1.13  2.07±1.15 | 1.70±0.79  1.33±1.02  2.00±0.83  1.47±1.16  2.00±1.02 | 0.949  0.391  0.742  0.900  0.803 |
